# Tropoelastin and Elastin Assembly

**DOI:** 10.3389/fbioe.2021.643110

**Published:** 2021-02-25

**Authors:** Jazmin Ozsvar, Chengeng Yang, Stuart A. Cain, Clair Baldock, Anna Tarakanova, Anthony S. Weiss

**Affiliations:** ^1^Charles Perkins Centre, The University of Sydney, Sydney, NSW, Australia; ^2^School of Life and Environmental Sciences, The University of Sydney, Sydney, NSW, Australia; ^3^Department of Biomedical Engineering, University of Connecticut, Storrs, CT, United States; ^4^Wellcome Trust Centre for Cell-Matrix Research, Division of Cell-Matrix Biology and Regenerative Medicine, Faculty of Biology, Medicine and Health, School of Biological Sciences, Manchester Academic Health Science Centre, University of Manchester, Manchester, United Kingdom; ^5^Department of Mechanical Engineering, University of Connecticut, Storrs, CT, United States; ^6^Sydney Nano Institute, The University of Sydney, Sydney, NSW, Australia

**Keywords:** elastin, elastic fibers, tropoelastin, computational modeling, assembly

## Abstract

Elastic fibers are an important component of the extracellular matrix, providing stretch, resilience, and cell interactivity to a broad range of elastic tissues. Elastin makes up the majority of elastic fibers and is formed by the hierarchical assembly of its monomer, tropoelastin. Our understanding of key aspects of the assembly process have been unclear due to the intrinsic properties of elastin and tropoelastin that render them difficult to study. This review focuses on recent developments that have shaped our current knowledge of elastin assembly through understanding the relationship between tropoelastin’s structure and function.

## Elastic Fibers and Elastin

Elastic fibers are present in the extracellular matrix (ECM) of vertebrate tissues, such as the skin, lungs, cardiovascular system, cartilage, and tendons. They are ubiquitous across most vertebrates other than lower vertebrates such as species from the superclass *Agnatha* (jawless fish) (Debelle and Tamburro, 1999). Elastic fibers provide tissues with mechanical resilience, durability, and cell interactivity, which support a diverse range of specialized functionality.

Elastic fibers are composed of approximately 90% elastin, whilst the remaining components are primarily comprised of fibrillin glycoproteins (Mecham, 1991). Thus, elastin is responsible, in great part, for the properties of elastic fibers. The most crucial of these properties is the ability to undergo many stretch-recoil cycles whilst maintaining the structural and functional integrity of elastic tissues over an organism’s lifetime. The ability to stretch and recoil arises from the biochemical properties of elastin’s monomer, tropoelastin (discussed below). Additionally, elastin is remarkably durable as it is primarily deposited during prenatal development and childhood, and is rarely synthesized during adulthood. Its estimated half-life of 70 years (Shapiro et al., 1991) is due to its extensive cross-linking and high hydrophobicity, which render it resistant to degradation (Vrhovski and Weiss, 1998; Schrader et al., 2018; Hedtke et al., 2019).

## Tropoelastin

Elastin’s subunit, tropoelastin, is a soluble 60–70 kDa protein which has been intensely studied over the past three decades. Tropoelastin is a spring-like molecule that is extremely extensible prior to cross-linking. A single tropoelastin molecule can stretch up to eight times its resting length and has a Young’s modulus (tensile stiffness) of ∼ 3 kPa (Baldock et al., 2011) in comparison to elastin’s extensibility of 150% and stiffness of ∼1 mPa (Aaron and Gosline, 1981). Tropoelastin undergoes minimal energy loss during extension, similar to other polymers such as rubber and resilin (Elvin et al., 2005; Cordier et al., 2008; Baldock et al., 2011). These remarkable properties arise from its sequence and structure, which render tropoelastin structurally highly flexible but not disordered (Tarakanova et al., 2018). In addition to its involvement in molecular elasticity, the flexibility of tropoelastin also a key requirement for self-assembly into elastin, with mutations that perturb this having detrimental effects on tissue (Yeo et al., 2016, 2017). Together, these two properties can be tuned and exploited to give rise to an increasing number of novel biomaterials for tissue engineering and regenerative medicine, which have been recently reviewed elsewhere (Wang et al., 2020; Wen et al., 2020).

## Tropoelastin Gene and Expression

Tropoelastin is encoded by the ELN gene, which is present in all vertebrates except jawless fish (Chung et al., 2006). Most organisms contain one copy of ELN, other than teleosts and amphibians which notably possess two distinct types of ELN genes (He et al., 2007; Miao et al., 2007, 2009). The human ELN gene is located on the long arm of chromosome 7q11.2 and comprises of 34 exons nestled between lengthy introns (Indik et al., 1987, 1989; Bashir et al., 1989). Human ELN gives rise to a broad variety of splice isoforms, with alternative splicing being observed with exons 22, 23, 24, 26A, 32, and 33 (Fazio et al., 1988; Parks et al., 1992) that result in 13 known human isoforms of the mature tropoelastin protein (Reichheld et al., 2019). The most commonly investigated isoform contains domain 26A and lacks domain 22, and is predominantly found in elastic tissues (Indik et al., 1987; Vrhovski et al., 1997).

Variations in the relative abundance of alternatively spliced ELN mRNA transcripts have been observed between tissues, and this diversity is thought to be necessary for the fine tuning of the mechanical characteristics of tissues to suit their unique functional requirements (Reichheld et al., 2019). Indeed, studies examining the consequences of domain insertions and deletions note changes in the intrinsic functionality of tropoelastin, corroborating the hypothesis that domain insertions and deletions result in altered tissue mechanics (Jensen et al., 2000; Kozel et al., 2003; Yeo et al., 2016; Miao et al., 2017). The isoform-function theory is further reinforced by the presence of two different ELN genes within teleosts that are differentially expressed both spatially and temporally during development (Miao et al., 2007). The self-assembly properties and nanostructure of these extra teleost isoforms are yet to be investigated.

## Sequence

**FIGURE 1:**
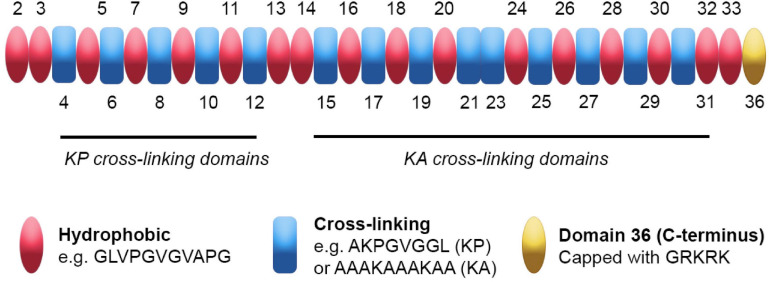
Tropoelastin’s sequence and domain arrangement. Tropoelastin is a low complexity protein on both primary and secondary sequence levels. Its hydrophobic (pink) and cross-linking (blue) domains consist of repetitive motifs that contribute uniquely to elastin assembly. The hydrophobic domains contain aliphatic amino acids with proline variations that provide flexibility and the ability to assemble into higher order structures. The cross-linking domains are enriched for either Lys-Pro (KP) or Lys-Ala (KA) motifs and form cross-links that link growing tropoelastin chains during elastogenesis; note that exon 6 encodes a KA domain. Tropoelastin’s C-terminal domain 36 (yellow) does not fall into either category as it contains a distinct sequence capped with a Gly-Arg-Lys-Arg-Lys (GRKRK) motif and is primarily involved in cell interactions.

Tropoelastin’s domains each arise from single exons of the ELN gene. They can be categorized as either “hydrophobic” or “cross-linking” based on their functionality and amino acid content (Figure 1). Tropoelastin’s primary sequence is low in complexity and contains repetitive motifs. Furthermore, tropoelastin’s hydrophobic and cross-linking domains are arranged in alternating patterns throughout the majority of the molecule, giving rise to both inter- and intra-domain level repetition.

Tropoelastin’s amino acid sequence predominantly consists of non-polar residues including glycine, valine, alanine, and proline (Debelle and Tamburro, 1999). The hydrophobic domains contain repetitions and variations of Val-Pro-Gly-Val-Gly motifs (Figure 1), resulting in the aforementioned low complexity of sequence (Foster et al., 1973; Gray et al., 1973). The hydrophobic domains vary in length, with the majority of the shorter (9–5 residues) domains occurring near the N-terminus, while the longer (up to 55 residues) domains are found within tropoelastin’s central and C-terminal regions (Indik et al., 1989). The hydrophobic domains have been extensively studied and are responsible for facilitating tropoelastin’s ability to self-assemble (Vrhovski et al., 1997; Toonkool et al., 2001).

The cross-linking domains are defined by lysines, which are responsible for the formation of durable bi-, tri-, and tetrafunctional cross-links within mature elastin. Cross-linking domains can be subdivided into KP or KA domains, which denote the amino acids (proline or alanine, respectively) that are adjacent to the lysines (Figure 1). KP domains are found closer toward the N-terminus, whereas KA domains are closer to the C-terminus and include alanine tracts (Indik et al., 1987). Unlike hydrophobic domains, cross-linking domains in isolation are unable to self-assemble; thus, they have been studied in the context of the cross-linking of elastin-derived biomaterials (Annabi et al., 2017; Yue et al., 2017). Cross-linking domains are capable of modulating self-assembly when incorporated into peptides and biomaterials derived from tropoelastin’s hydrophobic domains. For example, their inclusion in peptides derived from hydrophobic domains confers decreased times taken for self-assembly, most likely due to their favorable interaction with aqueous solvent (Miao et al., 2003). Modulative effects are also observed within full length tropoelastin, where disruptions to domain 26 diminish self-assembly (Jensen et al., 2000), highlighting the context of primary sequence on higher order structures.

Another region of note is domain 36, which contains lysines but does not participate in cross-linking (Hedtke et al., 2019). The amino acid sequence of domain 36 is unique; furthermore, its sequence confers a positively charged C-terminus comprising lysines between positively charged arginines, forming a RKRK sequence (Vrhovski et al., 1997). In addition to the RKRK sequence, domain 36 contains tropoelastin’s sole two cysteines and only disulfide bond. Perturbation of either of these components greatly reduces tropoelastin’s ability to self-assemble *in vitro* and interact with the microfibril scaffold of elastic fibers (Nonaka et al., 2014), indicating that an intact domain 36 is required for correct assembly.

## Structure

The understanding of tropoelastin’s structure has been hindered by numerous intrinsic properties of the molecule. Of most importance, the inability to acquire large quantities of pure tropoelastin historically presented a significant obstacle to experimentally characterizing its structure. Elastin is extensively cross-linked and difficult to break apart into monomers even under harsh conditions, thus, initial strategies included feeding animals a copper deficient diet to prevent lysyl oxidase (LOX) cross-linking of tropoelastin; however, this proved inefficient and time consuming (Wise and Weiss, 2009; Tarakanova et al., 2018).

Even after being able to produce recombinant tropoelastin at scales suited to characterization studies, traditional techniques have not yet produced an experimentally verified atomistic structure (Martin et al., 1995; Tarakanova and Buehler, 2013). Tropoelastin’s flexibility does not allow it to pack into a crystal lattice and its size renders it too complex to assign atomic interactions using nuclear magnetic resonance (NMR) on a global level (Tamburro et al., 2003, 2006; Bochicchio et al., 2004; Pepe et al., 2008). Cryo-electron microscopy is a rapidly developing method and likely to contribute to structural knowledge of tropoelastin. Indeed, cryo-EM was recently reported to have a lower molecular size limit of approximately 50 kDa, however, it has not yet been successfully applied to characterize tropoelastin (Murata and Wolf, 2018).

Due to the lack of a full-atomistic structure, a number of elastin derivatives, including isolated tropoelastin domains (Toonkool et al., 2001; Mackay et al., 2005; Dyksterhuis et al., 2007; Dyksterhuis and Weiss, 2010) and synthetic elastin-derived peptides (EDPs) (Luan et al., 1990; Tamburro et al., 1992; Kentsis and Sosnick, 1998; Kumashiro et al., 2006; Reichheld et al., 2014, 2017; Muiznieks et al., 2015; MacEwan et al., 2017; Tarakanova et al., 2017) have been studied to understand tropoelastin’s domain level properties. Attempts to characterize tropoelastin’s structure have seen a gradual shift from the belief that tropoelastin’s domains have fixed structures, to understanding that the majority of its domains are capable of transitioning between random coils and transient ordered structures. These ordered structures can include α-helices and β-structures (Vrhovski et al., 1997), which depend on the amino acid content and arrangement of domains (He et al., 2012; Reichheld et al., 2014, 2017; Muiznieks et al., 2015) and the choice of solvent (Reiersen and Rees, 2000; Muiznieks et al., 2015).

Tropoelastin’s hydrophobic domains are predominantly responsible for the random coil content of the monomer, and are the result of the unique pairing of proline and glycine pairs that are interspersed throughout the majority of hydrophobic domains (Rauscher and Pomes, 2017). The smallest amino acid, glycine is known to promote flexibility within local structures due to lack of steric hindrance, whereas proline’s restrictive sidechain inhibits conformational sampling and disrupts the formation of prolonged secondary structures (Roberts et al., 2015). Combinations of these two amino acids result in domains that undergo rapid conformational sampling (Rauscher and Pomes, 2017), which, if perturbed toward a conformation that gravitate toward stability, will assemble into highly ordered amyloid-like fibrils that are not elastomeric (Rauscher et al., 2006; Roberts et al., 2015).

More surprisingly, tropoelastin’s cross-linking domains are also capable of similar transitions between ordered and disordered structures. KA domains were initially thought to form α-helices and poly-proline II helices (PPII) due to the presence of cross-links which require a lysine arrangement that was postulated to be achieved via α-helical configuration (Brown-Augsburger et al., 1995). Although alanine tracts within other proteins are predisposed to α-helix formation (Yang and Honig, 1995; Avbelj, 2000), high helical content within tropoelastin’s KA domains was primarily demonstrated to persist in solvents that stabilize secondary structure, such as trifluoroethanol, rather than aqueous solution (Luo and Baldwin, 1997; Tamburro et al., 2006). More recently, it has been demonstrated that KA domains consist of random coil content prior to self-assembly and become more ordered as molecules aggregate, marrying the observations discussed above into a cohesive model within the context of assembly and the requirements of higher-order structures (Reichheld et al., 2014).

The first successful experiments to define the 3D envelope of tropoelastin utilized small angle X-ray scattering (SAXS) and small-angle neutron scattering (SANS) (Baldock et al., 2011). Tropoelastin was revealed to be an asymmetric molecule with distinct N- and C-terminal regions that are respectively referred to as the “head” and “foot” of the molecule. The N-terminal head caps an extended coil region that consists of domains 2–18. Below the coil is a flexible “hinge” region made up of domains 20–24, which are directly adjacent to the “bridge” regions of domains 25–26. Domains 27–36 make up tropoelastin’s foot, which are labeled as such due to their spatial arrangement, forming an almost claw-like component of the molecule. However, higher resolution data were required to map out its elusive properties and performance within a hierarchical assembly (Tarakanova et al., 2018; Ozsvar et al., 2019).

## Computational Models of Tropoelastin

**FIGURE 2:**
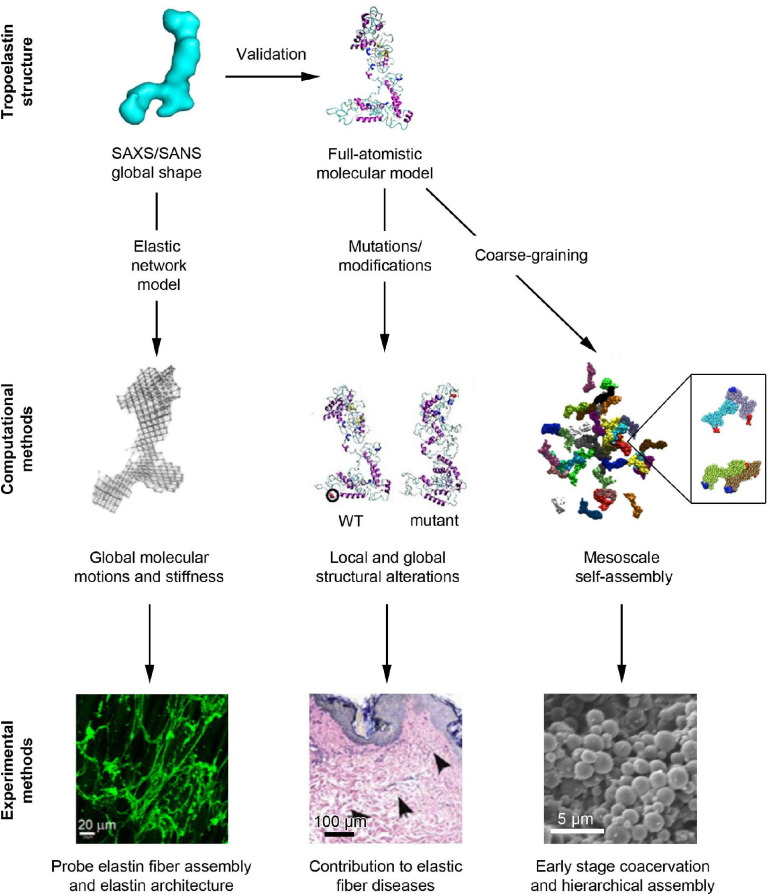
Overview of the computational and experimental methodologies that have recently contributed to our understanding of elastic fiber assembly. The SAXS/SANS global shape of tropoelastin (Baldock et al., 2011) has been used to validate the full-atomistic computational model of tropoelastin through a geometric and topological comparison (Tarakanova et al., 2018). Furthermore, the SAXS/SANS structure has been mapped to an elastic network model with tunable stiffness to probe the role of tropoelastin’s flexibility in fiber assembly (Yeo et al., 2016). Meanwhile, modifications to the full-atomistic model have revealed the mechanisms that contribute to aberrant fiber structure (Tarakanova et al., 2018) that have been hypothesized to predispose patients to diseases such as acquired cutis laxa (Hu et al., 2006). Additionally, coarse-graining the full-atomistic model has allowed for the examination of mesoscale tropoelastin assembly and, in particular, deciphered the orientation of tropoelastin molecules that occurs during early stage assembly (inset image) (Tarakanova et al., 2019a). Future investigations will allow the bridging of the gap between mesoscale simulations and microscopically observed coacervation (Clarke et al., 2006).

Computational approaches have gained popularity in recent decades as their methodologies have been refined to provide accurate atomistic scale insights into molecular structure and movement (Tarakanova et al., 2018). Thus, computational approaches can be considered as important means to complement wet bench experiments. By leveraging the advantages of computational models, such as elastic network models, full-atomistic models and coarse grained models, as depicted in Figure 2, the correlation between the structure, motions, and the functionality of tropoelastin have recently been explored in depth (Yeo et al., 2016; Tarakanova et al., 2018, 2019a).

The full-atomistic model of tropoelastin was developed based on replica exchange molecular dynamics (REMD) simulations, an accelerated sampling method for molecular dynamics (Tarakanova et al., 2018). This model revealed that tropoelastin maintains a canonical or “average” structure based on the distribution of its possible conformations in spite of its flexible nature (Figure 2), as well as the possible roles that local structures play in biological processes, specifically, elastogenesis (Tarakanova et al., 2019b). This canonical computational structure was determined to be highly similar to the envelope found via SAXS/SANS, where both consist of an extended molecular body accompanied by a protruding foot (Baldock et al., 2011). The computational model uncovered the contributions of each molecular region to the flexibility of the molecule. For example, the highly flexible domains 2–5 that were noted to generate a twisting motion in N-terminus, were positioned beside domain 6, a relatively immobile region that may assist in stabilizing the subsequent regions. The flexibility of the mid-region of the molecule was proposed to drive the overall elasticity of the resultant fibers. Closer to the base of the molecule, the flexible hinge region between domains 21 and 23 presented with a scissors-like bending, which is now believed to contribute to elasticity and multimeric assembly through enhanced conformational space sampling (Tarakanova et al., 2019b). Further down the molecule, the cell-interactive C-terminus was noted to be highly flexible, indicating that high conformational sampling could be conducive to tropoelastin’s interactions between cell-surface receptors and elastic fiber-associated proteins. Thus, the current computational model unifies the global and regional characteristics of tropoelastin, inferring mechanisms that complement observed experimental phenomena.

Computational models have also been leveraged to pinpoint the molecular consequences of synthetic and disease mutations of tropoelastin. For example, models involving single point mutations at negatively charged residues demonstrated both regional and global destabilization of tropoelastin’s structure, which were validated by SAXS structures (Yeo et al., 2012; Tarakanova et al., 2018). The reduction in solvent accessible surface area of the mutant molecules suggests that the underlying mechanism for their altered self-assembly properties observed in experiments is due to less exposure of the appropriate hydrophobic domains required for coacervation. Similarly, a mutation model associated with cutis laxa, or “loose skin disease” (Hu et al., 2006), was found to exhibit higher stability compared with the wild-type molecule due to the increased longevity of its salt bridges (Hu et al., 2006; Tarakanova et al., 2018). The resultant bending motion – rather than scissors-twist motion – that stemmed from this stabilization highlights the mutant’s diminished flexibility as a key driver of impaired fiber assembly in cutis laxa (Tarakanova et al., 2018). By probing mutations in computational studies that resolve the atomistic structure of the molecule, the deduction of mechanisms associated with changes to functional roles of specific domains has become feasible, and a connection between local structures and biological performance can be established (Figure 2; Tarakanova et al., 2018).

Stiffness within the context of self-assembly has also been examined with both coarse grained and full-atomistic simulations using polypeptides derived from tropoelastin’s hydrophobic domains. The stiffness that arose due to amino acid substitutions partially determined resultant secondary structure which, in turn, impacted assembly (Prhashanna et al., 2019). Such models could be used in conjunction with the full molecular model of tropoelastin to examine the self-assembly of discrete regions to understand the impacts of amino acid substitutions in disease. Unsubstituted models are also critical to understanding self-assembly. Modeling utilizing tropoelastin derivatives has demonstrated that structural compaction into both coils and globules occurs above the transition temperature of self-assembly, which may assist in explaining the anisotropic nature of mature elastin (Baul et al., 2020).

The importance of tropoelastin’s flexibility during self-assembly has also been examined in the light of natural allysine modifications, which condense to give cross-links within mature elastin (discussed further in the following section) (Ozsvar et al., 2019). The conversion of a single lysine to an allysine results in structural stabilization, which may serve as a checkpoint during self-assembly to ensure that molecules incapable of forming multiple cross-links are not incorporated into the growing chain. This deduction has been corroborated by the observation that tropoelastin with multiple allysine modifications displays conformational sampling comparable to that of the wild-type molecule, suggesting it is more likely to readily participate in self-assembly.

## Elastogenesis

Elastogenesis is the term that collectively describes the hierarchical process of elastic fiber formation, and is comprised of distinct phases: tropoelastin synthesis, coacervation, cross-linking, and deposition.

**FIGURE 3:**
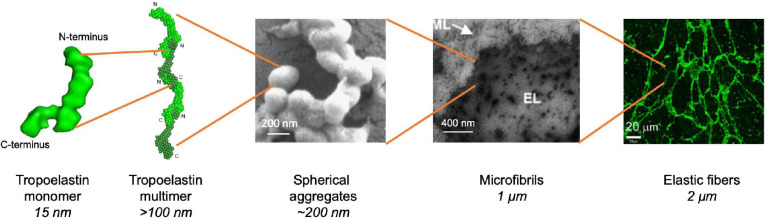
Stages of hierarchical assembly of elastic fibers. Tropoelastin monomers undergo self-assembly upon reaching the transition temperature through the aggregation of their hydrophobic domains (Wise et al., 2014). Assembly proceeds from a nucleation event and undergoes elongation in a step-wise manner to form a multimer which can occur in a head-to-tail fashion (Wise et al., 2014). Multimers may undergo further transitions, such as branching, to form spherules made of multimer aggregates (Tu et al., 2010). The spherules grow in size and are deposited onto the microfibril scaffold where they fuse into fibrillar structures (Sherratt et al., 2001). Elastic fibers are eventually formed after extensive cross-linking through a process termed maturation (Yeo et al., 2016).

Elastogenic cells, such as fibroblasts (Mecham et al., 1985) and smooth muscle cells (Narayanan et al., 1976), synthesize and secrete tropoelastin. The majority of tropoelastin synthesis occurs during perinatal development (Myers et al., 1983; Noguchi et al., 1990), however, synthesis may be triggered in response to tissue damage (Parks et al., 1992) or during diseases such as atherosclerosis (Phinikaridou et al., 2018). Tropoelastin is secreted as a ∼60–70 kDa protein to the cell surface where it participates in self-assembly (Hinek and Rabinovitch, 1994; Figure 3).

Coacervation is an endothermic, entropically favorable process through which tropoelastin monomers self-assemble into higher order n-mer structures. Coacervation optimally occurs at physiological temperature, however, *in vitro* coacervation can also occur at lower temperatures depending on experimental conditions and the choice of tropoelastin isoform or derivative (Yeo et al., 2011). This temperature is also known as the transition temperature. Initially, *in vitro* coacervation is characterized by the rapid aggregation of tropoelastin. into 200 nm then 1–2 μm spherules, which eventually grow and stabilize into spherules 2–6 μm in diameter (Clarke et al., 2006; Kozel et al., 2006; Tu and Weiss, 2010; Tu et al., 2010). Tropoelastin spherules assemble at the cell surface before deposition onto the microfibrillar scaffold in cell culture systems at physiological temperature (Kozel et al., 2006). The process of tropoelastin aggregation is initially reversible, as spherules dissipate if the temperature is lowered (Clarke et al., 2006), however, maintenance of a physiological temperature results in maturation, which is indicated by spherule coalescence and the irreversible formation of fibrillar structures (Cox et al., 1974; Bressan et al., 1983; Mithieux et al., 2005). The presence of tropoelastin spherules fusing to fibrils has been noted in native tissue, demonstrating marked similarities between *in vitro* and *in vivo* coacervation (Haust et al., 1965; Albert, 1972; Kozel et al., 2006).

Tropoelastin’s hydrophobic domains are primarily responsible for facilitating coacervation (Tamburro et al., 1992; Miao et al., 2003; Muiznieks et al., 2003). Non-polar residues are a major contributor to protein folding, as their unfavorable interactions with water drive them to bury into the protein core, however, as tropoelastin is comprised of numerous hydrophobic domains, it has been demonstrated that many of these domains will be at least partially solvent exposed (Dyksterhuis et al., 2007). Thus, at lower temperatures, the water surrounding these domains forms ordered, clathrate-like shells that prevent aggregation until the appropriate temperature is reached (Wu and Weiss, 1999; Miao et al., 2003; Dandurand et al., 2015). In contrast, higher temperatures allow the breaking of the hydrogen bonds of the ordered water, dissipating the clathrate shells and permitting the association of the hydrophobic domains (Yeo et al., 2011). The prevention of early self-aggregation *in vitro* is thought to be mediated by chaperone proteins (Hinek and Rabinovitch, 1994; Miao et al., 2013). Further to this, the flexibility of the hydrophobic domains may also play a key role in self-assembly. Molecular dynamics modeling of aggregating tropoelastin-derived peptide chains points to the maintenance of a hydrated, disordered, liquid-like state due to the formation of short-lived inter-chain bonds (Rauscher and Pomes, 2017; Reichheld et al., 2020), mostly likely due to the inducement of random coils by PG repeats (*vide supra*). This phenomenon is supported by *in vitro* peptide studies, which note that increasing the space between PG motifs or removing prolines results in more ordered structures (Rauscher et al., 2006).

Similar to other ECM proteins, such as collagen, tropoelastin covalently cross-links via its lysines. Approximately 90% of tropoelastin’s lysines undergo modification and/or participate in cross-links, indicating that mature elastin is extensively cross-linked (Kozel et al., 2003; Schmelzer et al., 2019). Cross-linking requires the modification of at least one of the lysine participants by a member of the copper-containing LOX or lysine oxidase-like (LOXL) enzyme families. LOX and LOXL convert the ε-amino group of lysine to α-aminoadipic acid δ-semialdehyde (allysine) (Schmelzer et al., 2019), which spontaneously undergo either a Schiff base reaction with a lysine, or two allysines crosslink through aldol condensation, to give rise to bifunctional crosslinks (Franzblau et al., 1969; Lent et al., 1969). The bifunctional cross-links can undergo further condensation to form tetrafunctional desmosine or isodesmosine (Partridge, 1966). A series of mapping studies have been recently conducted to pinpoint the locations of these cross-links (Schrader et al., 2018; Hedtke et al., 2019; Schmelzer et al., 2019) as their placements are crucial to understanding the resultant molecular orientation of tropoelastin within cross-linked elastin. Molecular docking studies utilizing tropoelastin and the 3D structure of LOX1 (Vallet et al., 2018) may assist in our understanding as to whether the enzyme has a preference for modifying particular tropoelastin residues.

The next stage of elastin assembly involves the deposition of tropoelastin spherules onto the microfibril scaffold of elastic fibers. Microfibrils comprise multiple proteins, of which fibrillin-1 is the most common. Tropoelastin interacts with microfibril components including fibrillin-1, fibulin-4, and -5, and other associated molecules such as latent transforming growth factor β binding protein-4 (Visconti et al., 2003; McLaughlin et al., 2006; Urban et al., 2009; Yamauchi et al., 2010; Noda et al., 2013; Lockhart-Cairns et al., 2020). It has recently been suggested that fibrillins are capable of stabilizing tropoelastin, possibly for the purpose of selecting conformations that are favorable for elastin assembly (Lockhart-Cairns et al., 2020). In addition to interacting with tropoelastin, fibulin-4 and -5 are capable of also binding LOX and fibrillin-1 and, thus, have key roles in facilitating elastogenesis (Hinderer et al., 2015). Moreover, fibulins are essential for elastic fiber directionality, as fibroblasts with fibulin-4 and -5 knockdowns generate poorly formed elastic fibers (Yamauchi et al., 2010), and fibulin-4 –/– mice display aberrant, poorly cross-linked, and non-fibrous elastin (McLaughlin et al., 2006).

## Current Model of Tropoelastin Assembly

It was first hypothesized that tropoelastin assembles in a head-to-tail manner similar to that of other ECM proteins such as collagen, which assembles into fibrils that subsequently associate laterally to form sheets and thicker fibers (Kadler, 2017). Solving tropoelastin’s global structure (Baldock et al., 2011) allowed for 3D mapping on tropoelastin of the approximate locations of domains 10, 19, and 25, which were the first unequivocally cross-linked domains to be discovered (Brown-Augsburger et al., 1995). The alignment of these three domains indicates that at least two tropoelastin molecules are required to form this tetrafunctional cross-link, thus, giving rise to the head-to-tail model of assembly involving the growth of a linear chain that can assemble laterally to form sheets and eventually form spherules (Baldock et al., 2011).

However, there are aspects of this model that warrant further investigation. For example, the tetrafunctional cross-link, on which the model is based, is derived from porcine elastin (Brown-Augsburger et al., 1995). Although there is no 3D structure for porcine tropoelastin, porcine tropoelastin differs from that of humans (Sandberg et al., 1977), leaving it unclear as to whether these domains align as expected in human tissue. Further to this, the current model of elastin assembly does not account for how long strings or sheets of tropoelastin are capable of forming spherules on a macroscopic scale (Tu et al., 2010).

More recently, the computational model of tropoelastin has been leveraged to characterize higher-order structures and probe self-assembly (Tarakanova et al., 2019a). Forty tropoelastin molecules were subjected to simulations using a MARTINI-based coarse-grained model, integrated with an elastic network model (Tarakanova et al., 2019a). The advantage of coarse-grained methodologies is that they allow for the simulation of timescales of large molecular systems on the order of microseconds, which are otherwise computationally infeasible with classical full-atomistic molecular dynamics. Dominant driving factors of assembly were examined, including water, temperature and domain pair orientations. Crucially, these simulations revealed that self-assembly starts with a nucleation event and elongation proceeds via both globular and fibrillar structures (Tarakanova et al., 2019a). This suggests a high level of conformational sampling during this phase of coacervation, indicating that the flexibility of tropoelastin plays a key role in assembly that may persist, to some extent, into later stages of assembly (Reichheld et al., 2020). Importantly, the presence of fibrils indicates that the nanostructures formed during initial assembly contribute to the supramolecular structures that arise during both early (spherule) and later (fibril) stages of elastogenesis. Interestingly, the location and orientation of different domains that contacted each other during self-assembly was noted to be heterogeneous, resulting in head-to-head, tail-to-tail, head-to-tail, and lateral interactions (Tarakanova et al., 2019a). This reflects the experimentally observed heterogeneity of cross-links in mature elastin, again giving weight to the hypothesis that the flexibility of tropoelastin is imperative for assembly (Schrader et al., 2018; Tarakanova et al., 2019a).

## Tropoelastin-Cell Receptor Interactions and Implications for Assembly

Tropoelastin promotes cell attachment and migration of several cell types including fibroblasts, endothelial cells and mesenchymal stem cells. Cellular activities are mediated through interactions between tropoelastin or EDPs and specific receptors on the cell surface. These interactions trigger a wide range of processes including wound healing, elastogenesis and maintenance of stemness (Yeo and Weiss, 2019).

Elastin binding protein (EBP) is a splice variant of β-galactosidase, that recognizes the repetitive hydrophobic sequences (VGVAPG) of tropoelastin (Tajima et al., 1997). EBP plays two roles in the assembly of elastin. The first is an intracellular role as a tropoelastin chaperone and the second is as part of the elastin receptor complex (ERC). Intracellularly, EBP is associated with tropoelastin after the release of the signal peptide and acts as a chaperone to prevent self-aggregation and proteolysis as it transported to the cell membrane, after which EBP is then recycled and serves as a reusable shuttle protein (Hinek et al., 1995).

On the cell surface, EBP forms a complex with protective protein/cathepsin A (PPCA) and neuraminidase-1 (Neu-1), giving rise to the ERC (Duca et al., 2007). The ERC binds elastin derived peptides, which are the product of proteolytic activity of soluble and insoluble elastin by various elastases. Elastases can be in the form of serine- (e.g., Ela-2), cysteine- (e.g., cathepsin I), or matrix metalloproteinases (MMP-2, -7, -9, and -12), and bioactive peptides include the VGVAPG peptide. Binding of EDPs to the ERC induces a number of biological effects including migration, adhesion, proliferation, protease expression, and secretion. EDPs can modulate an array of signal pathways (Scandolera et al., 2016), but full-length tropoelastin does not trigger pathways through the ERC. This suggests the ERCs’ primary signal pathways are involved in wound recognition through fragments of elastin, where EDPs are formed as a result of elastic tissue damage.

Glycosaminoglycans (GAGs) are negatively charged, linear polysaccharides, which can be either sulfated [heparan sulfate (HS) or chondroitin sulfate] or non-sulfated (hyaluronic acid). HS, which is present on cell surface proteoglycans (syndecans and glypicans), interacts strongly with tropoelastin (Cain et al., 2005). Positively charged lysine residues in domains 17 were identified to interact with negatively charged HS, most likely via ionic interactions (Lee et al., 2017). The last 17 amino acid residues of the C-terminus of domain 36 are also implicated in HS interactions; the last four residues are Arg-Lys-Arg-Lys and are positively charged at physiological pH which provides a charged cluster capable of binding negatively charged HS (Broekelmann et al., 2005). It has been postulated that the interaction between tropoelastin and cell surface proteoglycans is part of the assembly process of elastin before it is deposited on microfibrils. Fibrillin-1 and -2 also interact with proteoglycans (Tiedemann et al., 2001), which coupled with tropoelastin/GAG interactions provide a pathway to the formation of elastic fibers.

Integrins are a major class of cell surface receptor family, of which tropoelastin has been found to interact with two types: α_*v*_β_3_ (Rodgers and Weiss, 2004) and α_*v*_β_5_. Integrins canonically bind proteins containing Arg-Glu-Asp motifs, but this sequence is not found in tropoelastin (Lee et al., 2014). Instead, tropoelastin domains 14–18 and 36 (RKRK sequence) have been found to bind to both integrins. The lysines of domain 15 and 17 are believed to play key roles in this interaction. It is likely that, as a general rule, integrin binding to tropoelastin is mediated via GAGs, which is facilitated by the repetition of tropoelastin’s primary sequence and the surface exposure of positively charged similar domains (Lee et al., 2017; Bochicchio et al., 2021). Interactions with these integrins on the surface of mesenchymal stem cells, either through surface bound or as soluble tropoelastin, promote MSC proliferation and phenotypic maintenance via FAK and PKB/AKT (Yeo and Weiss, 2019). The narrow specificity to α_*v*_β_3_ and α_5_β_3_ may also be beneficial compared with other ECM adhesion molecules such as fibronectin (which can adhere to up to 20 types of integrin) which may compete with and modulate binding to tropoelastin, and deliver opposing effects on cell proliferation. Furthermore, as integrins are involved in the remodeling of the ECM (Bonnans et al., 2014), this is consistent with a model where the tropoelastin-integrin interaction forms part of the wound repair process.

## Conclusion

Tropoelastin is a unique protein with biochemical and physical properties that allow it to rapidly self-assemble into fibrous structures. It has become increasingly evident that tropoelastin’s sequence, structure and subsequent functionality are in a delicate balance; thus, perturbations to tropoelastin’s sequence can have far-reaching consequences for self-assembly and the resultant architecture of elastic fibers. For many years it was difficult to study tropoelastin at an atomic scale, but the application of computational methods, such as full-atomistic molecular dynamics and elastic network models, in combination with powerful low-resolution structural studies, have expanded the field and delivered an enhanced understanding of the mechanisms that contribute to self-assembly. Modeling has been verified using wet-bench methodologies, forming a robust suite of complementary methodologies that will undoubtedly become more prevalent for exploring the assembly of biological fibers over time. With leaps in the improvement of cryogenic electron microscopy to characterize flexible molecules, we predict that this approach will contribute to a deeper understanding of tropoelastin structure and self-assembly in the context of endogenous fiber formation and biomaterials fabrication.

## Author Contributions

All authors contributed to writing and editing the review.

## Conflict of Interest

AW is the Scientific Founder of Elastagen Pty. Ltd., which was sold to Allergan, now a division of AbbVie. The remaining authors declare that the research was conducted in the absence of any commercial or financial relationships that could be construed as a potential conflict of interest.
